# Exogenous melatonin ameliorates drought stress in *Agropyron mongolicum* by regulating flavonoid biosynthesis and carbohydrate metabolism

**DOI:** 10.3389/fpls.2022.1051165

**Published:** 2022-12-19

**Authors:** Jing Wang, Xueqin Gao, Xing Wang, Wenxue Song, Qin Wang, Xucheng Wang, Shuxia Li, Bingzhe Fu

**Affiliations:** ^1^School of Agriculture, Ningxia University, Yinchuan, Ningxia, China; ^2^Ningxia Grassland and Animal Husbandry Engineering Technology Research Center, Yinchuan, Ningxia, China; ^3^Key Laboratory for Model Innovation in Forage Production Efficiency, Ministry of Agriculture and Rural Affairs, Yinchuan, Ningxia, China

**Keywords:** *Agropyron mongolicum*, melatonin, drought stress, flavonoid biosynthesis, carbohydrate metabolism

## Abstract

Drought is one of the most common abiotic stressors in plants. Melatonin (MT) is a high-efficiency and low-toxicity growth regulator that plays an important role in plant responses to drought stress. As a wild relative of wheat, *Agropyron mongolicum* has become an important species for the improvement of degraded grasslands and the replanting of sandy grasslands. However, the physiological and molecular mechanisms by which exogenous MT regulates drought stress in *A. mongolicum* remain unclear. To assess the effectiveness of MT intervention (100 mg·L^−1^), polyethylene glycol 6000 was used to simulate drought stress, and its ameliorating effects on drought stress in *A. mongolicum* seedlings were investigated through physiology, transcriptomics, and metabolomics. Physiological analysis indicated that MT treatment increased the relative water content and chlorophyll content and decreased the relative conductivity of *A. mongolicum* seedlings. Additionally, MT decreased malondialdehyde (MDA) and reactive oxygen species (ROS) accumulation by enhancing antioxidant enzyme activities. The transcriptome and metabolite profiling analysis of *A. mongolicum* seedlings treated with and without MT under drought stress identified the presence of 13,466 differentially expressed genes (DEGs) and 271 differentially expressed metabolites (DEMs). The integrated analysis of transcriptomics and metabolomics showed that DEGs and DEMs participated in diverse biological processes, such as flavonoid biosynthesis and carbohydrate metabolism. Moreover, MT may be involved in regulating the correlation of DEGs and DEMs in flavonoid biosynthesis and carbohydrate metabolism during drought stress. In summary, this study revealed the physiological and molecular regulatory mechanisms of exogenous MT in alleviating drought stress in *A. mongolicum* seedlings, and it provides a reference for the development and utilization of MT and the genetic improvement of drought tolerance in plants from arid habitats.

## Introduction

*Agropyron mongolicum* is a diploid perennial herb of Poaceae. It has significant value for studying the origin, evolution, and genetic diversity of species, and it is an important grass in forage production and ecological construction (Che and Li, 2007). The ecological range of *A. mongolicum* is very broad, and it is mainly distributed in the desert steppe of central-western China. As a high-quality forage grass in grassland areas, the hay yield of *A. mongolicum* occupies an important position among the grasses of a typical steppe (Liu et al., 2013). In addition, as a wild relative species of wheat, *A. mongolicum* is not only an important resistance gene pool for wheat breeding but is also an important species for degraded grassland improvement and sandy grassland reseeding (Zhao et al., 2018).

With increasing global temperature, drought stress is becoming one of the major threats to the growth and development of plants (Rollins et al., 2013). Drought stress causes intricate physiological and biochemical reactions in plant cells by disturbing water balance and reducing plant water-use efficiency. Plants respond to drought stress mainly by increasing the content of antioxidants, low molecular weight osmolytes, and plant growth substances (Farooq et al., 2012). However, excessive drought in a short period or prolonged exposure to drought conditions can cause irreversible cell damage, eventually leading to cell death. Therefore, it is necessary to explore some alternative exogenous drought-resistant chemicals to enhance the intrinsic drought-resistant actions, thereby mitigating the adverse effects of drought on plant production.

Melatonin (MT), an indoleamine compound, was first discovered in animals (Lerner et al., 1958). In the 1990s, MT was detected in higher plants and then extensively used in the plant field (Hattori et al., 1995; Manchester et al., 2000; Ramakrishna et al., 2012). MT has multiple functions in the process of plant growth and development, including promoting photosynthesis (Zhao et al., 2015), participating in lateral root formation (Bajwa et al., 2014), delaying leaf senescence (Liang et al., 2015), regulating flower development and fruit ripening, and regulating the circadian rhythm (Sun et al., 2015), even if its content is extremely low. Additionally, MT is well-known for its unique antioxidant properties, and it acts as an important defender for plants against oxidative stress (Wang et al., 2018; Debnath et al., 2019). Many studies have demonstrated that exogenous MT can improve plant tolerance to abiotic stresses, such as ultraviolet radiation, low temperature, high temperature, drought, salinity, and alkali ions (Zhu et al., 2019; Cen et al., 2020; Guo et al., 2020; Yao et al., 2021). Recently, considerable research has been performed to decipher the functions of exogenous and endogenous melatonin in the drought stress mitigation of plants. These studies indicated that the pretreatment of melatonin through foliar spray, seedling treatment, and root dipping regulates physiological processes such as osmoregulation, germination, antioxidant mechanisms, photosynthesis, ion homeostasis, antisenescence primary and secondary metabolism, and hormonal cross-talks in plants under water deficit conditions (Cui et al., 2017; Li et al., 2018a). In addition, studies on the molecular mechanism of MT in alleviating drought stress in plants showed that MT enhances drought tolerance in plants mainly by regulating the MAPK pathway, photosynthetic system, flavonoid biosynthesis, ascorbate-glutathione cycle, hormone metabolism, and carbohydrate metabolism (Liang et al., 2018; Campos et al., 2019; Ibrahim et al., 2020; Moustafa et al., 2020; Hu et al., 2022). For example, exogenous MT application can alleviate the damage caused by drought stress on maize (Ren et al., 2021), cotton (Hu et al., 2022), loquat (Wang et al., 2021), and soybean (Cao et al., 2021) by regulating starch and sucrose metabolism. Furthermore, MT can enhance drought tolerance in citrus (Jafari and Shahsavar, 2021), grape (Deluc et al., 2009), and soybean (Cao et al., 2020) by promoting flavonoid biosynthesis. However, the regulatory mechanism of MT in *A. mongolicum* under drought stress has not yet been reported.

In this study, we analyzed the effects of 100 mg·L^−1^ MT on *A. mongolicum* under drought stress through physiology, transcriptomics, and metabolomics to provide a reference for the utilization of MT and the genetic improvement of drought tolerance in plants from arid habitats.

## Materials and methods

### Plant material and experimental design

*Agropyron mongolicum* ‘Yanchi’ is a new forage variety bred by Ningxia University after several years of selection and domestication. Seeds of *A. mongolicum* were disinfected with 10% sodium hypochlorite (*v/v*) for 10 min. Subsequently, the seeds were rinsed thoroughly with distilled water. The seeds were then placed in petri dishes (9 cm in diameter) with two layers of filter paper, and 50 seeds were placed in each petri dish after adding 5 mL of distilled water. Afterwards, the petri dishes were placed in an artificial climate incubator under the following conditions: temperature of 25°C/20°C (day/night), humidity of 55–65%, photoperiod of 14 h light/10 h dark, and light intensity of 1000 μmol m^−2^ s^−1^. Water was replenished every day to maintain moisture, and the filter paper was changed every 3 days. After 12 days, the seedlings with the same morphology were transferred to Hoagland nutrient solution for hydroponics, and the nutrient solution was replaced every 5 days. When the seedlings were hydroponically cultivated for 28 days, the leaves were sprayed with distilled water or MT solution under pretreatment for 7 days; each pot was sprayed with 50 mL of 100 mg·L^−1^ MT or distilled water every day. After pre-spraying for 7 days, drought stress was simulated with 12% polyethylene glycol 6000 (PEG) solution. The concentrations of 100 mg·L^−1^ MT and 12% PEG were chosen based on a preliminary study. Thus, four treatments were included in this study: (i) control (CK), Hoagland nutrient solution plus foliar pre-spray for 7 days with distilled water; (ii) melatonin (M), Hoagland nutrient solution plus foliar pre-spray for 7 days with 100 mg·L^−1^ MT; (iii) drought stress (D), Hoagland nutrient solution containing 12% PEG plus foliar pre-spray for 7 days with distilled water; (iv) drought stress with melatonin (MD), Hoagland nutrient solution containing 12% PEG plus foliar pre-spray for 7 days with 100 mg·L^−1^ MT. Each treatment has three biological replicates for a total of 75 seedlings, which are uniformly distributed in the pots (20 × 14 × 13 cm). Water was replenished every 2 days, and the PEG concentration remained constant. *A. mongolicum* seedling leaves were collected on the 1st, 3rd, 5th, and 7th days after treatment for the physiological experiment. The leaves were sampled on the 7th day of treatment and immediately stored at −80°C for transcriptomic and metabolomic analysis.

### Measurement of physiological indicators

Plant height was measured with a ruler. The number of wilted leaves in each treatment was counted to calculate the leaf wilt rate. The relative water content was estimated following Ahmad et al. (2019). The relative conductivity was estimated according to the methods of a previous study (Wu et al., 2017). Chlorophyll content was determined *via* spectrophotometry (Arnon, 1949). The malondialdehyde (MDA) content was determined using the thiobarbituric acid method (Puckette et al., 2007). Superoxide anion radical (O_2_^.-^) content was determined by the p-aminobenzenesulfonic acid method, and hydrogen peroxide (H_2_O_2_) content was spectrophotometrically determined as described by Okuda et al. (1991). Superoxide dismutase (SOD) was determined using the nitroblue tetrazolium method (Niu et al., 2017), and peroxidase (POD) activity was measured using the methoxyphenol method (Wang et al., 2013). The proline (Pro) content was determined using the acid-ninhydrin method (Bates et al., 1973). Three biological replicates were performed for each of the above indicators.

### RNA extraction and sequencing

The RNA samples were extracted from the leaves of *A. mongolicum* plants under different treatments. Each treatment was represented by three biological replicates of the leaf samples. Total RNA was extracted from leaf tissues using an RNAprep Pure Plant kit (Tiangen, Beijing, China). RNA quality was detected by a NanoPhotometer spectrophotometer (IMPLEN, CA, USA), Qubit 2.0 Fluorometer (Life Technologies, Carlsbad, CA, USA), and an Agilent Bioanalyzer 2100 system (Agilent Technologies, Santa Clara, CA, USA). The cDNA (~200 bp) was screened using AMPure XP beads. After amplification and purification, cDNA libraries were obtained and sequenced using the Illumina HiSeqTM 2000 system (Illumina, San Diego, CA, USA).

The raw data were filtered using fastp v 0.19.3, and all subsequent analyses were based on clean reads. Transcriptome assembly was performed using Trinity (v2.11.0). Gene expression levels were estimated using RSEM, and the fragments per kilobase of transcripts per million (FPKM) value of each gene was calculated based on the gene length. Differential expression analysis between the two groups was performed using DESeq2 v1.22.1. A |log_2_Fold Change| ≥ 1 and FDR < 0.05 were used as the threshold for significant differential expression. Gene Ontology (GO) and Kyoto Encyclopedia of Genes and Genomes (KEGG) enrichment analyses were performed based on a hypergeometric test.

### Metabolites analysis by LC-MS/MS

The freeze-dried samples were ground into a powder and extracted with 70% aqueous methanol. After centrifugation at 10,000×g for 10 min, all supernatants were combined and filtered through a 0.22-mm pore size membrane and then processed for UPLC-ESI-MS/MS analysis (UPLC, SHIMADZU Nexera X2, https://www.shimadzu.com.cn/; MS, Applied Biosystems 4500 Q TRAP, https://www.thermofisher.cn/cn/zh/home/brands/applied-biosystems.html). Three biological replicates were set for each treatment in metabolites analysis. The effluent was alternatively connected to an ESI-triple quadrupole linear ion trap (Q TRAP)-MS. Linear ion trap (LIT) and triple quadrupole (QQQ) scans were acquired on a triple quadrupole-linear ion trap mass spectrometer equipped with an ESI Turbo Ion-Spray interface operating in positive ion mode and controlled by Analyst 1.6.3 software (AB Sciex). A scheduled multiple reaction monitoring method was used to quantify the metabolites.

Significantly regulated metabolites between groups were determined by VIP ≥ 1 and log_2_|FoldChange| > 1. The identified metabolites were annotated using the KEGG Compound database (http://www.kegg.jp/kegg/compound/). Annotated metabolites were then mapped to the KEGG Pathway database (http://www.kegg.jp/kegg/pathway.html). Pathways with significantly regulated metabolites were then input into metabolite set enrichment analysis (MSEA); their significance was determined by p-values from a hypergeometric test. Principal component analysis (PCA; base package), correlation analysis using R (base package; Hmisc), and plot heatmaps (ComplexHeatmap) were performed using R software.

### Quantitative real-time RCR analyses

For qRT-PCR, RNA was extracted using a Trizol Total RNA Extraction Kit (Sangon Biotech, Shanghai, China) and reverse transcribed using an Evo M-MLV RT Mix Kit with gDNA Clean (Accurate Biotechnology, Hunan, China). The primers are listed in **Supplementary Table 1**. qRT-PCR was conducted using a BioEasy Master Mix (SYBR Green) Kit (Bioer, Hangzhou, China) and a C1000 TouchChihermal Cycler system (Bio-Rad). All tested transcripts were normalized to the reference gene *TLF*, and relative transcript levels were calculated according to the 2^-ΔΔCt^ method (Tian et al., 2016). Three biological and technical replications were performed.

### Statistical analysis

The data were analyzed with Excel 2019 (Microsoft Inc., Redmond, USA) and SPSS 22 (SPSS Inc., Chicago, USA) using an analysis of variance (ANOVA), followed by Duncan’s significant difference test at *P* < 0.01 or *P* < 0.05. The graphs were constructed using Origin 2020 (Electronic Arts Inc., San Francisco, CA, USA).

## Results

### Physiological analysis of MT-treated *A. mongolicum* seedlings under drought stress

To determine whether MT improved the drought tolerance of *A. mongolicum*, PEG and MT treatments were performed on the seedlings, and phenotypic and physiological traits were analyzed. The preliminary experiments for PEG concentration screening (0, 3, 6, 9, 12, and 15% PEG) showed that under 12% PEG, the changes of each index were larger, and the leaf wilt rate was close to 50%, indicating that the use of 12% PEG solution to simulate drought stress had a significant but not serious inhibitory effect on *A. mongolicum* seedlings. Therefore, this concentration was selected as the concentration of drought treatment in subsequent experiments (**Supplementary Figures 1 and 2A–I**). The preliminary experiments for MT concentration screening (0, 1, 10, 50, 100, 150, and 200 mg·L^−1^ MT) showed that 100 mg· L^−1^ MT treated *A. mongolicum* had a significantly higher plant height, dry aboveground weight, relative water content, and chlorophyll content compared with plants under drought stress; thus, this concentration was selected for the MT treatment in subsequent experiments (**Supplementary Figures 3 and 4A–I**).

**Figure 1:**
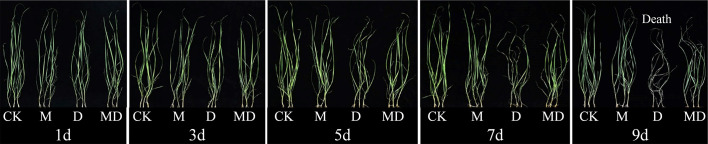
Effects of 100 mg·L^−1^ MT on the phenotype of *A. mongolicum* seedlings under different durations of drought stress.

**Figure 2:**
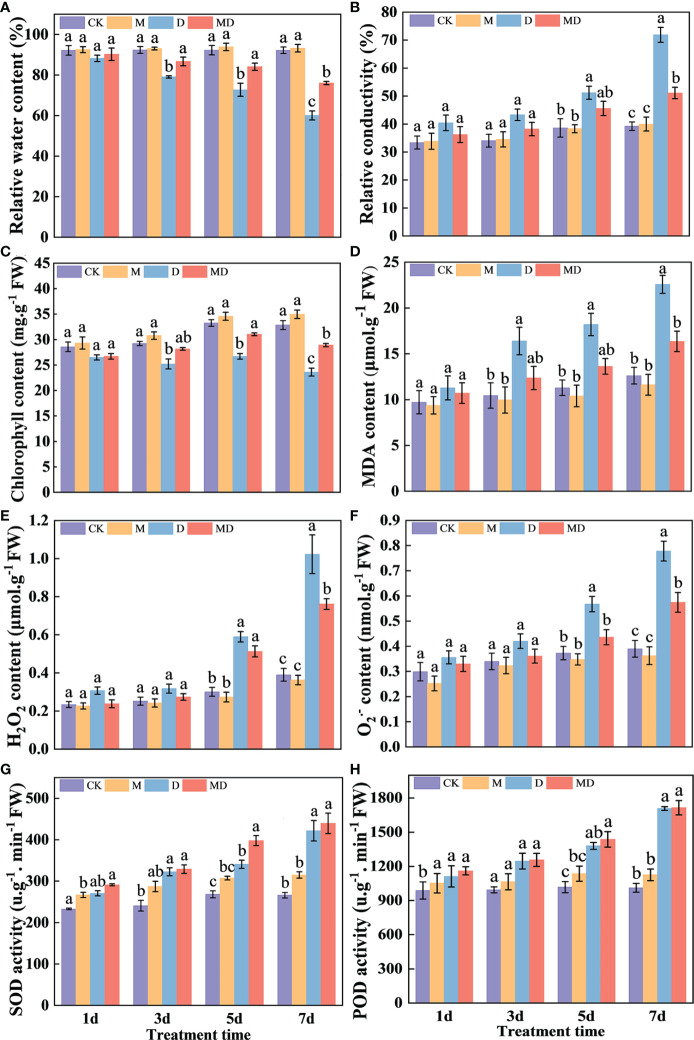
Effects of 100 mg·L^−1^ MT on the physiology of *A. mongolicum* seedlings under different durations of drought stress. **(A)** Relative water content, **(B)** relative conductivity, **(C)** chlorophyll content, **(D)** MDA content, **(E)** H_2_O_2_ content, **(F)** O_2_^−^ content, **(G)** SOD activity, **(H)** POD activity. Mean values followed by different letters were significantly different by Duncan’s test (*P* < 0.01).

Based on the optimal PEG and MT concentrations screened, we analyzed the effects of exogenous MT addition on the physiology of *A. mongolicum* seedlings under different drought stress times. MT treatment significantly alleviated the withering of seedling leaves under drought stress. After 7 days of drought stress, although the leaves of seedlings wilted in both the MD and D treatments, the degree of wilting in the MD treatment was lower than that in the D treatment. After 9 days of drought stress, the leaves under D treatment withered and died (**Figure 1**). On the 1st day of drought stress, all physiological indexes showed no significant difference between treatments. Compared with the control, drought stress significantly decreased the relative water content and chlorophyll content of leaves of *A. mongolicum* seedlings on the 7th day of drought stress. However, MT application dramatically mitigated this effect (**Figures 2A, C**). MT treatment significantly decreased the relative conductivity and MDA content under drought stress but had no obvious effect on the control plants (**Figures 2B, D**). In addition, drought treatment resulted in a significant increase in H_2_O_2_ and O_2_^.-^ contents, while MT addition significantly decreased their content (**Figures 2E, F**). Drought treatment enhanced SOD and CAT activities, and there was no significant difference in them after MT addition (**Figures 2G, H**). The results showed that the *A. mongolicum* seedlings at 7 days directly reflected a turning point in the degree of drought stress. Therefore, we selected the *A. mongolicum* leaves of CK, M, D and MD treatments at 7 days (serious drought) to perform transcriptomic and metabolomic analysis.

### Transcriptome analysis of MT-treated *A. mongolicum* seedlings under drought stress

To explore the molecular mechanism of MT regulating the drought resistance of *A. mongolicum* seedlings, transcriptome analysis was performed on *A. mongolicum* seedlings treated with and without MT under drought stress. After removing the low-quality reads, a total of 86.19 Gb clean data were obtained. The percentage of GC was 49.01–57.44%, and the Q30 bases of each sample were above 92.63%, indicating that the data quality and purity of transcriptome sequencing were high. A total of 149,051 genes were functionally annotated in at least one database (**Supplementary Table 2**). Moreover, 10,293 (5140 upregulated and 5153 downregulated) and 13,466 (6670 upregulated and 6796 downregulated) differentially expressed genes (DEGs) were identified in the comparison groups of “CK vs. D” and “D vs. MD,” respectively (**Supplementary Table 3**). These results indicate that MT induced changes in the transcripts of *A. mongolicum* seedlings under drought stress.

### DEG analysis of MT-treated *A. mongolicum* seedlings under drought stress

**Figure 3:**
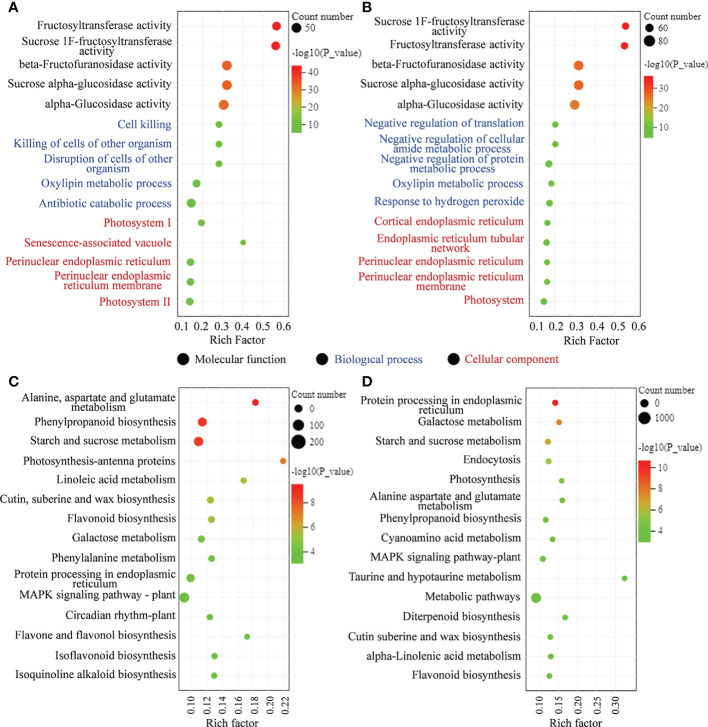
DEGs enriched on different GO terms and KEGG pathways. **(A)** GO terms of DEGs in “CK vs. D.” **(B)** GO terms of DEGs in “D vs. MD.” **(C)** KEGG pathway analysis of DEGs in “CK vs. D.” **(D)** KEGG pathway analysis of DEGs in “D vs. MD.”.

To further clarify the response to MT, the biological functions, GO and KEGG enrichment analyses of DEGs were performed in MT-treated *A. mongolicum* seedlings under drought stress (**Supplementary Table 4**). The GO terms of DEGs in “CK vs. D” and “D vs. MD” were significantly enriched in “fructosyltransferase activity,” “sucrose 1F-fructosyltransferase activity,” “beta-fructofuranosidase activity,” “sucrose alpha-glucosidase activity,” and “alpha-glucosidase activity” in molecular function classes (**Figure 3A**). In addition, the GO terms of DEGs in “CK vs. D” were mainly enriched in “cell killing,” “killing of cells of other organism,” and “disruption of cells of other organism” in biological process classes and were enriched in “photosystem I” and “senescence-associated vacuole” in cellular composition classes. The GO terms of DEGs in “D vs. MD” were mainly enriched in “negative regulation of translation” and “negative regulation of cellular amide metabolic process” in biological process classes and were enriched in “cortical endoplasmic reticulum” and “endoplasmic reticulum tubular network” in cellular composition classes (**Figures 3A, B**).

KEGG enrichment analysis showed that several pathways related to plant stress resistance or metabolism were enriched in “CK vs. D” and “D vs. MD,” including galactose metabolism, starch and sucrose metabolism, phenylpropanoid biosynthesis, flavonoid biosynthesis, protein processing in endoplasmic reticulum, and alanine, aspartate, and glutamate metabolism (**Figures 3C, D**).

### Metabolome analysis of MT-treated *A. mongolicum* seedlings under drought stress

**Figure 4:**
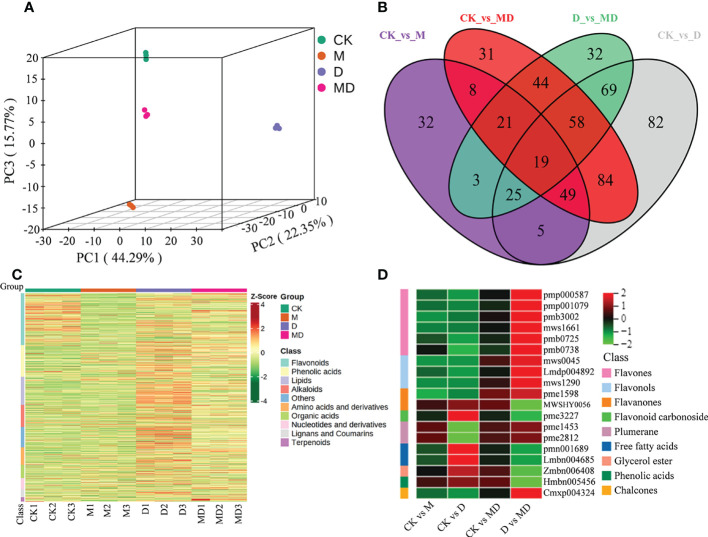
Metabolites analysis of *A. mongolicum* seedlings under drought stress. **(A)** PCA analysis of metabolites. **(B)** Venn diagram of DEMs. **(C)** Heat map visualization of metabolites. Red indicates high abundance, whereas relatively low-abundance metabolites are shown in green (color key scale at the right of the heat map). **(D)** Heat map analysis of common DEMs in “CK vs. MD,” “CK vs. D,” “CK vs. MD,” and “D vs. MD.”.

To elucidate the specific effects on metabolites, we carried out broadly targeted metabolome assays. PCA showed that the three biological replicates for each treatment were clustered together and clearly divided into four groups, indicating significant metabolic differences between treatments. In addition, the metabolism of these four different treatments was significantly separated in the first component (PC1), except for CK and M treatments, indicating that the effect of MT on the metabolism of *A. mongolicum* under drought stress was obvious (**Figure 4A**). All identified metabolites are presented in **Supplementary Table 5**. The number of metabolites in “CK vs. M,” “CK vs. D,” “CK vs. MD,” and “D vs. MD” was 162, 391, 314, and 271, respectively (**Figure 4B** and **Supplementary Table 6**). These differentially expressed metabolites (DEMs) were mainly classified into flavonoids (261), phenolic acids (160), lipids (142), alkaloids (110), amino acids and derivatives (88), organic acids (67), nucleotides and derivatives (56), lignans and coumarins (40), terpenoids (21), and others (103) (**Figure 4C**). Interestingly, among the 19 DEMs involved in “CK vs. M,” “CK vs. D,” “CK vs. MD,” and “D vs. MD,” flavones and flavonols were highly accumulated in “D vs. MD” but not in “CK vs. MD” and “CK vs. D.” However, the DEMs of flavanones, flavonoid carbonoside, free fatty acids, gycerol ester, and phenolic acids were all significantly upregulated in “CK vs. D,” but downregulated in “D vs. MD” (**Figure 4D** and **Supplementary Table 7**). MT induced changes in antioxidants in *A. mongolicum* seedlings under drought stress.

### Integrated analysis of genes and metabolites related to flavonoid biosynthesis in MT-treated *A. mongolicum* under drought stress

Based on the integrated analysis of the transcriptome and metabolome, we focused on flavonoid biosynthesis, galactose metabolism, and starch and sucrose metabolism pathways (**Supplementary Table 8**).

**Figure 5:**
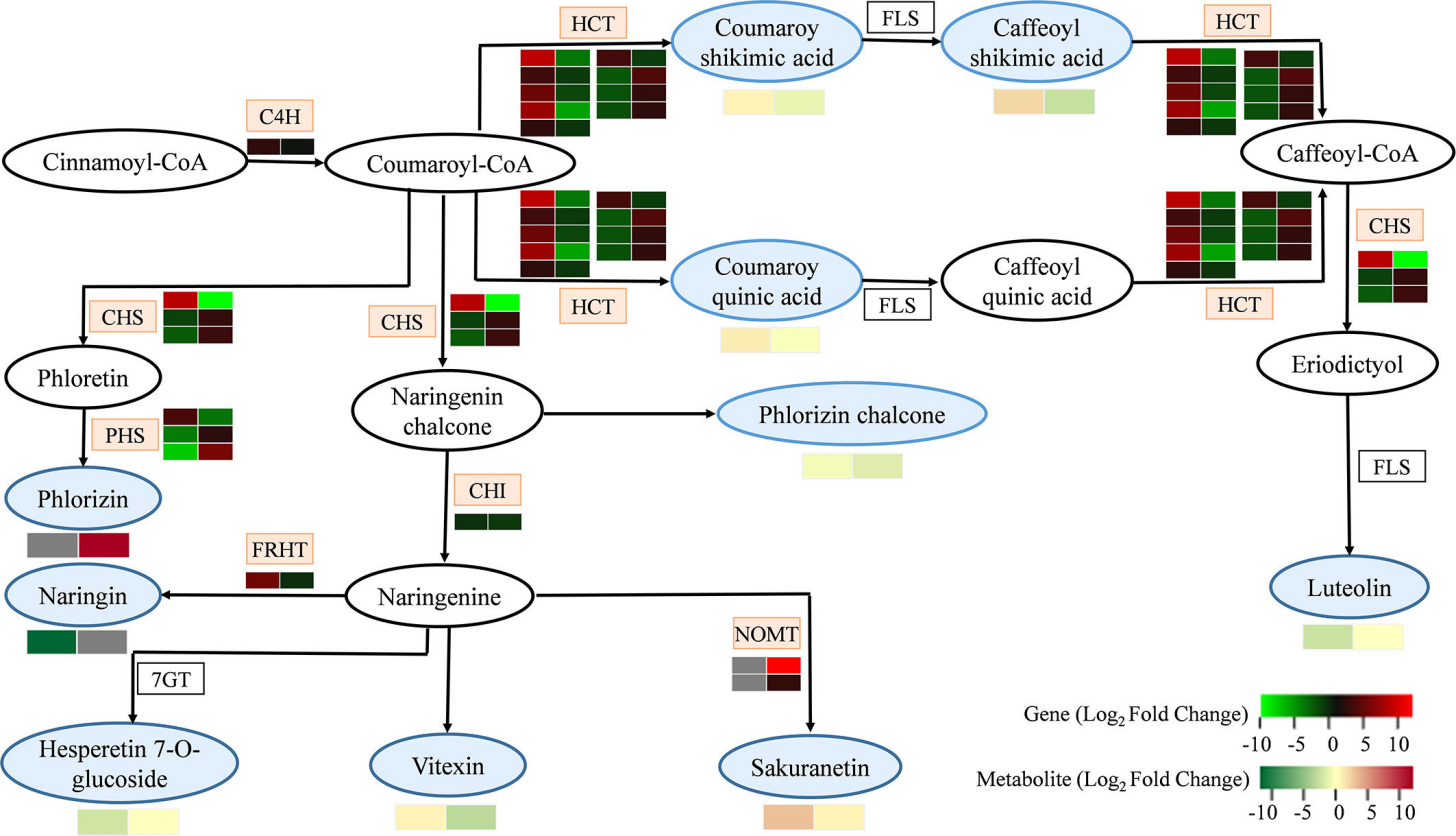
DEGs and DEMs involved in flavonoid biosynthesis in response to exogenous addition of MT under drought stress. The blue and pink patterns represent the metabolites and genes that changed after exogenous addition of MT under drought stress, respectively. The rectangle is divided into two equal parts: the left of the rectangle represents DEGs or DEMs in “CK vs. D”; the right of the rectangle represents DEGs or DEMs in “D vs. MD.” The colors in the rectangle represent the genes or metabolites regulated after exogenous addition of MT under drought stress (red indicates upregulation; green indicates downregulation; gray indicates undetected).

To explore the impacts of MT on secondary metabolism in *A. mongolicum* under drought stress, the interaction of DEGs and DEMs related to flavonoid biosynthesis was analyzed (**Figure 5** and **Supplementary Table 9**). We found that three DEGs encoding chalcone synthase (CHS), three DEGs encoding phlorizin synthase (PHS), and nine DEGs encoding shikimate O-hydroxycinnamoyl transferase (HCT) were differentially expressed in “CK vs. D,” and the expression patterns of these DEGs in “D vs. MD” were opposite to that in “CK vs. D.” One gene encoding cinnamate-4-hydroxylase (C4H) was significantly upregulated in both “CK vs. D” and “D vs. MD,” and the DEG encoding chalcone isomerase (CHI) was significantly downregulated. Furthermore, two DEGs encoding naringenin 7-O-methyltransferase (NOMT) were significantly upregulated in “D vs. MD,” but they were not detected in “CK vs. D.” Moreover, in the flavonoid biosynthesis pathway, sakuranetin and coumaroyl quinic acid accumulated in both “CK vs. D” and “D vs. MD,” but phlorizin accumulated only in “D vs. MD.”

**Figure 6:**
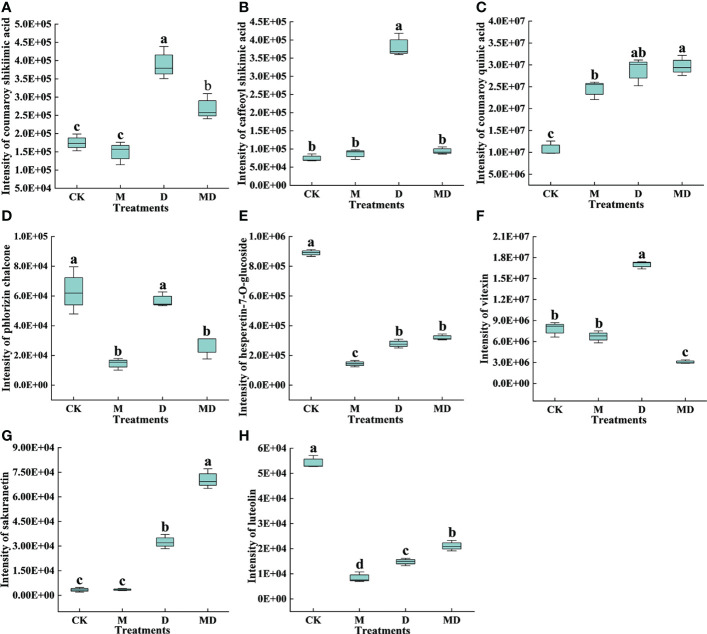
Effects of exogenous MT on DEMs in the flavonoid biosynthetic pathway in *A. mongolicum* leaves under drought stress. **(A)** Coumaroyl shikimate acid, **(B)** caffeoyl shikimic acid, **(C)** coumaroyl quinic acid, **(D)** phlorizin chalcone, **(E)** hesperetin-7-O-glucoside, **(F)** vitexin, **(G)** sakuranetin, **(H)** luteolin. Error bars represent standard errors of triplicate experiments. Mean values with different letters are significantly different at *P* < 0.05.

As shown in **Figures 6A–H**, three metabolites, namely coumaroy shikimic acid, caffeoyl shikimic acid, and vitexin, in flavonoid biosynthesis were more abundant under D treatment compared to CK, and they were less abundant in MD treatment than in D treatment. The content of hesperetin-7-O-glucoside and luteolin in CK was significantly higher than those in M, D, and MD treatments. The content of coumaroyl quinic acid and sakuranetin was significantly higher in the D and MD treatments than in CK. Moreover, the phlorizin chalcone content in the CK and D treatments was significantly higher than that in the D and MD treatments. The DEGs and DEMs related to flavonoid biosynthesis in MT-treated *A. mongolicum* were considered to conjointly respond to drought stress.

### Integrated analysis of genes and metabolites related to carbohydrate metabolism in MT-treated *A. mongolicum* under drought stress

**Figure 7:**
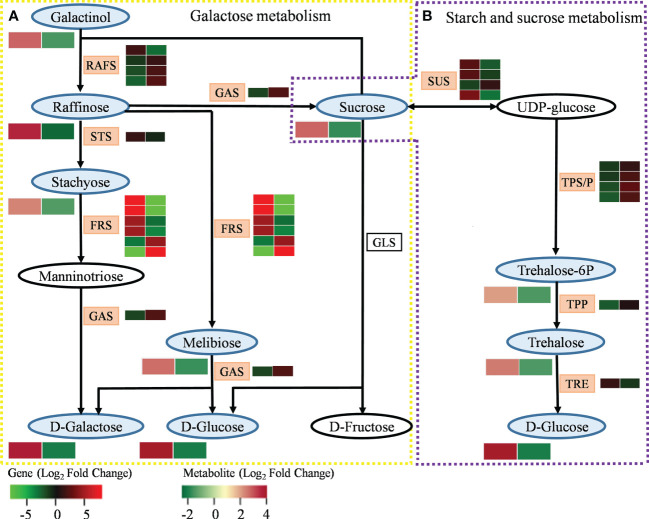
DEGs and DEMs involved in carbohydrate metabolism in response to exogenous addition of MT under drought stress. **(A)** Galactose metabolism. **(B)** Starch and sucrose metabolism. The blue and pink patterns represent the metabolites and genes that changed after exogenous addition of MT under drought stress, respectively. The rectangle is divided into two equal parts: the left of the rectangle represents DEGs or DEMs in “CK vs. D”; the right of the rectangle represents DEGs or DEMs in “D vs. MD.” The colors in the rectangle represent the genes or metabolites regulated after exogenous addition of MT under drought stress (red indicates upregulation; green indicates downregulation; gray indicates undetected).

Analyzing the interaction of DEGs and DEMs related to galactose metabolism enables us to better understand the complex mechanism of MT in the galactose metabolism of *A. mongolicum* under drought stress (**Figure 7A** and **Supplementary Table 10**). A DEG encoding raffinose synthase (RAFS) was upregulated in “CK vs. D,” while three DEGs encoding RAFS were downregulated. Four DEGs encoding beta-fructofuranosidase (FRS) were upregulated in “CK vs. D,” while two DEGs encoding FRS were downregulated. The expression patterns of 10 DEGs encoding RAFS and FRS were opposite in “CK vs. D” and “D vs. MD.” The DEGs encoding stachyose synthetase (STS) were significantly upregulated in “CK vs. D” but were downregulated in “D vs. MD.” A gene encoding alpha-galactosidase (GAS) was significantly downregulated in “CK vs. D” but was significantly upregulated in “D vs. MD.” In addition, in the galactose metabolism pathway, the DEMs of galactinol, raffinose, stachyose, D-galactose, melibiose, D-glucose, and sucrose were significantly upregulated in “CK vs. D” but were downregulated in “D vs. MD.” The DEGs and DEMs related to galactose metabolism in *A. mongolicum* conjointly responded to drought stress after exogenous MT addition.

Starch and sucrose metabolism plays an important role in plant growth and development. Analysis of the interaction of DEGs and DEMs related to this pathway can further clarify the regulatory mechanism of MT to alleviate drought stress in *A. mongolicum* (**Figure 7B** and **Supplementary Table 11**). The three DEGs encoding sucrose synthase (SUS) were significantly upregulated, and one DEG was significantly downregulated in “CK vs. D.” The expression pattern of these DEGs in “D vs. MD” was opposite that in “CK vs. D.” The four DEGs encoding trehalose 6-phosphate synthase/phosphatase (TPS/P) and one DEG encoding trehalose 6-phosphate phosphatase (TPP) were significantly downregulated in “CK vs. D” but were upregulated in “D vs. MD.” One DEG encoding alpha, alpha-trehalase (TRE) was significantly upregulated in “CK vs. D” but was downregulated in “D vs. MD.” Among starch and sucrose metabolic pathways, the DEMs of sucrose, trehalose 6-phosphate, trehalose, and D-glucose were significantly upregulated in “CK vs. D” but were significantly downregulated in “D vs. MD.” The DEGs and DEMs related to the starch and sucrose metabolism of *A. mongolicum* conjointly responded to drought stress after exogenous MT addition.

**Figure 8:**
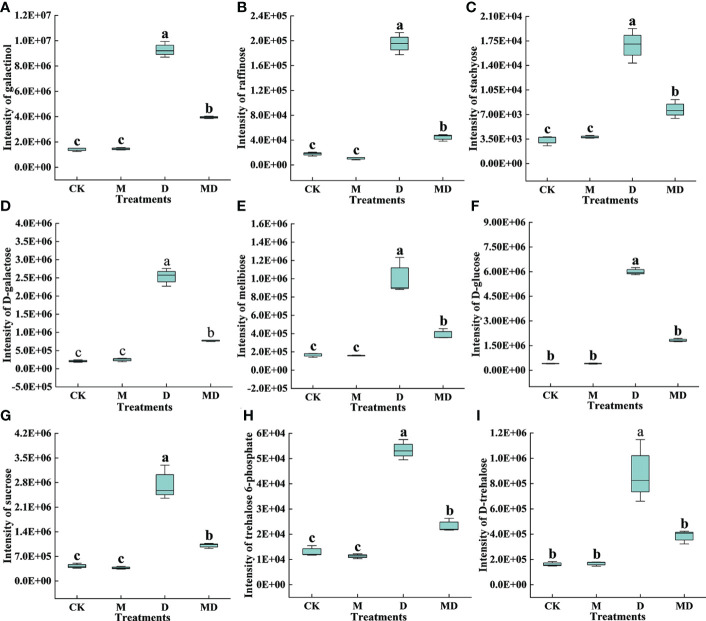
Effects of exogenous MT on DEMs in the carbohydrate metabolism pathway in *A. mongolicum* leaves under drought stress. **(A)** Galactinol, **(B)** raffinose, **(C)** stachyose, **(D)** D-galactose, **(E)** melibiose, **(F)** D-glucose, **(G)** sucrose, **(H)** trehalose 6-phosphate, **(I)** D-trehalose. Error bars represent standard errors of triplicate experiments. Mean values with different letters are significantly different at *P* < 0.05.

As shown in **Figures 8A–I**, based on the content of DEMs related to carbohydrate metabolism, nine metabolites (galactinol, raffinose, stachyose, D-galactose, melibiose, D-glucose, sucrose, trehalose 6-phosphate, and D-trehalose) were more abundant in the D treatment compared to the CK treatment, and they were less abundant in the MD treatment when compared to the D treatment.

### Correlation analysis of DEGs and DEMs in flavonoid metabolism and carbohydrate metabolism in MT-treated *A. mongolicum* seedlings under drought stress

**Figure 9:**
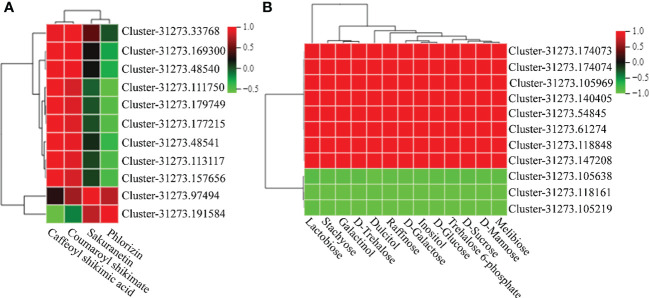
Correlation cluster heatmap of genes and metabolites. **(A)** Correlation cluster heatmap of DEGs or DEMs in flavonoid biosynthetic pathways. **(B)** Correlation cluster heatmap of DEGs or DEMs in carbohydrate metabolism pathways. The color in the box represents the correlation of genes and metabolites (red indicates positive correlation; green indicates negative correlation).

To examine the relationship between DEGs and DEMs in MT-treated *A. mongolicum* seedlings under drought stress, a correlation analysis of DEGs and DEMs involved in flavonoid and carbohydrate metabolism was conducted (|Pearson correlation coefficient| > 0.8, *P* value < 0.05) (**Figure 9** and **Supplementary Tables 12****,** **13**). In flavonoid metabolism, caffeoyl shikimic acid and coumaroyl shikimate were strongly positively correlated with genes encoding flavanone 7-O-glucoside 2’’-O-beta-L-rhamnosyltransferase (FRHT), HCT, PHS, and CHS. Meanwhile, sakuranetin and phlorizin were strongly positively correlated with the *C4H* and *NOMT* genes. In carbohydrate metabolism, lactobiose, stachyose, galactinol, D-trehalose, dulcitol, raffinose, D-galactose, inositol, D-glucose, trehalose 6-phosphate, D-sucrose, D-mannose, and melibiose were strongly positively correlated with genes encoding FRS, SUS, and TRE.

### qRT-PCR validation

To confirm the accuracy and reproducibility of the RNA-seq data, nine DEGs were selected from flavonoid biosynthesis, galactose metabolism, and starch and sucrose metabolism for qRT-PCR. RNA-seq (FPKM) and qRT-PCR analyses were in concordance (**Supplementary Figure 5**), showing the same general expression trends. These results verified the reproducibility and reliability of the RNA-seq data.

## Discussion

### Physiological mechanism by which MT alleviates drought stress in *A. mongolicum* seedlings

As one of the leading abiotic stressors, drought detrimentally affects plant morphology and physiology, thereby inhibiting the growth and development of plants (Liu et al., 2015). Our results show that drought stress delayed the growth and development of *A. mongolicum* seedlings by reducing the relative water content and chlorophyll content, accumulating ROS and MDA, which causes membrane lipid peroxidation, and ultimately disrupting the structure and function of the cell membrane. MT is known as a broad-spectrum antioxidant that scavenges ROS and increases antioxidant enzyme activity under drought stress (Reiter et al., 2016). Herein, we found that MT application increased the relative water content and chlorophyll content, decreased the accumulation of ROS and MDA, and further increased the activities of antioxidant enzymes in *A. mongolicum* seedlings exposed to drought stress (**Figures 2A–F**). These results suggest that drought stress is alleviated by MT and those of its metabolites that are free endogenous radical scavengers. Similarly, Antoniou et al. (2017) and Cui et al. (2017) reported that exogenous MT addition significantly facilitated the growth and development of alfalfa and wheat and alleviated the accumulation of intracellular ROS and MDA induced by drought stress by enhancing its ability to scavenge free radicals. Therefore, the exogenous addition of an appropriate concentration of MT can alleviate the growth inhibition of *A. mongolicum* seedlings under drought stress by affecting physiological effects.

### MT improved flavonoid biosynthesis in *A. mongolicum* seedlings under drought stress

Flavonoid biosynthesis is one of the most widely studied metabolic pathways (Mierziak et al., 2014). Many studies have shown that flavonoid metabolism is involved in plant response to drought stress (Yuan et al., 2012). Flavonoids are important secondary metabolites that can eliminate ROS accumulation through synergistic action with other stress response factors, such as MT and ABA, and ultimately improve the adaptability of plants to arid environments (Naghizadeh et al., 2019; Hossain et al., 2020; Yang et al., 2021b; Zou et al., 2021).

In the flavonoid biosynthesis pathway, the *C4H* gene controls the synthesis of p-coumaric acid from trans-cinnamic acid, which ultimately produces pigments (flavonoids) and plant defense compounds (furanocoumarins, isoflavonoids, and norlignans) (Suzuki et al., 2002; Suzuki et al., 2004). Drought stress caused an increase in the expression of the *C4H* gene in the leaves of *A. mongolicum* seedlings, and exogenous MT addition further enhanced the expression of the *C4H* gene under drought stress (**Figure 5**). Previous studies have also shown that C4Hs can enhance plant resistance to drought stress by increasing phenolic synthesis (Wang et al., 2017). Together, CHS and CHI constitute rate-limiting enzymes for flavonoid biosynthesis (Braune et al., 2016). Coumaroyl-CoA is catalyzed by CHS to form chalcone (Tan et al., 2015). Naringenin chalcone is an intermediate in flavonol biosynthesis and is converted to naringenin by CHI (Muir et al., 2001). In many plants, some external stimuli, such as adversity stress, induce the rapid response and expression of *CHS* and *CHI* (Muir et al., 2001). In this study, one *CHI* was significantly downregulated in both “CK vs. D” and “D vs. MD.” Two *CHS* genes were significantly downregulated in “CK vs. D” but were upregulated in “D vs. MD,” which indicated that MT induced the upregulation of two *CHS* genes and indirectly increased the flavonoid content, such as phloridzin and luteolin, and improved the antioxidant capacity of *A. mongolicum* (**Figure 5**). Previous studies have shown the complementary effects of phloridzin in dihydrochalcone compounds, which act as functional antioxidants to reduce oxidative stress in plants (Venisse et al., 2001). Natural flavonoids, such as luteolin, have the effect of scavenging free radicals (Wang et al., 2013) and can enhance the enzymatic activity of SOD, CAT, and other antioxidant enzymes (Lim et al., 2007; Choi et al., 2008). Song et al. (2022) showed that MT treatment greatly enhanced the stress tolerance of pigeon pea by promoting luteolin biosynthesis.

NOMT is a key enzyme in phytoalexin biosynthesis that catalyzes the methylation of naringenin to sakuratin (Tamogami et al., 1997). Rakwal et al. (2000) showed that NOMT was not found in the healthy tissues of rice or in the control treatment in our study. However, MT treatment significantly upregulated the expression of two *NOMT* genes under drought stress, thus enhancing the drought resistance of *A. mongolicum* seedlings by regulating the downstream metabolite sakuratin. In addition, HCT, a key enzyme in flavonoid biosynthesis, is required for coumaroyl-CoA to generate caffeoyl-CoA (Sun et al., 2018). *HCT* overexpression in pears leads to flavonoid accumulation that enhances their tolerance to drought stress (Yang et al., 2021a). In our study, six genes regulating HCT were significantly upregulated in “CK vs. D” but were significantly downregulated in “D vs. MD.” This was similar to the content of two intermediate metabolites of coumaroyl shikimate acid and caffeoyl shikimic acid in the process of p-coumaroyl-CoA producing caffeoyl-CoA. However, the content of some compounds, such as coumaroyl shikimate acid and caffeoyl shikimic acid, decreased after exogenous MT addition under drought stress, which may be due to the reduced accumulation of some flavonoids induced by MT, resulting in more energy conversion to cope with drought stress (Zou et al., 2021).

### MT enhanced carbohydrate metabolism in *A. mongolicum* seedlings under drought stress

Carbohydrate metabolism is one of the most important metabolic processes in plants. It plays an important role in the complex regulatory networks of hormones, reactive oxygen species (ROS) production and scavenging, energy storage, and signal transduction throughout the plant life cycle (Bolouri et al., 2010). As a plant bioregulator, exogenous MT can change the content of metabolites in the process of carbohydrate metabolism by regulating related genes and activating the activities of related enzymes, thereby enhancing the tolerance of plants to drought stress (Arnao et al., 2021).

In carbohydrate metabolism, RAFS is a key enzyme that catalyzes the galactinol production of raffinose. Studies have shown that raffinose content in plants increases significantly when subjected to adversity stress (Egert et al., 2013). In this study, a gene encoding RAFS was upregulated under drought stress, increasing the raffinose content (**Figure 7**). Stachyose is mainly used as an antioxidant to scavenge ROS accumulated under stress conditions. In the present study, a gene encoding STS was significantly upregulated under drought stress, resulting in a massive accumulation of stachyose in the leaves of *A. mongolicum*. In addition, four genes encoding FRS were significantly upregulated under drought stress, causing an increase in melibiose content. Khan et al. (2021) showed that the expression level of FRS increased after drought stress in citrus trees. However, after exogenous MT addition under drought stress, *RAFS STS* and four *FRSs* were downregulated, resulting in a decrease in the content of downstream metabolites raffinose, stachyose, and melibiose. In addition, GAS, as an exoglycosidase that catalyzes the hydrolysis of α-galactosidic bonds, is mainly involved in the hydrolysis of melibiose, raffinose, and stachyose. Enhanced GAS activity reduces melibiose, raffinose, and stachyose (Hu et al., 2016). The results of this study showed that after exogenous MT addition under drought stress, a gene encoding GAS was significantly upregulated, decreasing the content of upstream metabolites, such as melibiose, raffinose, and stachyose. Trehalose, as a non-reducing disaccharide, is synthesized by TPS and TPP (Holmström et al., 1996). The accumulation of trehalose in plant cells can improve drought tolerance (Romero et al., 1997). In this study, trehalose was significantly accumulated under drought stress, and then, it was decomposed into glucose under the action of TRE. However, under drought stress, the trehalose content decreased after exogenous MT addition, and the downregulated expression of a gene encoding TRE resulted in a decrease in the content of its downstream metabolite glucose, which may indicate that MT addition alleviated drought stress by reducing trehalose degradation.

SUS is a key enzyme that regulates sucrose synthesis (Liu et al., 2016). Drought stress typically induces increases in plant SUS activity, which in turn increases sucrose accumulation and promotes the activity of pathways related to carbohydrate metabolism, ultimately alleviating the damage caused by drought (Yang et al., 2001). This study showed that the expression levels of the three *SUS* genes and sucrose content increased under drought stress. However, after exogenous MT addition, the expression levels of the three *SUS* genes decreased, causing a decrease in sucrose content, which indicates that MT application might accelerate the decomposition of sucrose, resulting in a reduction in sucrose content (Li et al., 2018b). Wang et al. (2021) also showed that two *SUS* genes were downregulated after exogenous MT addition under drought stress. Therefore, we speculate that the decrease in carbohydrate content after exogenous MT addition under drought stress may be due to MT enhancing carbohydrate conversion and transport capacity under drought stress, and exogenous MT addition leads to a decrease in energy production by reducing carbohydrate metabolism, thus slowing plant growth and development to adapt to the stimulation of an arid environment.

## Conclusion

This study provides new insights into the mitigating effects of MT against drought stress in *A. mongolicum*. Based on the analysis of the physiology, transcriptome, and metabolome of *A. mongolicum* seedlings under drought stress by exogenous MT addition, the regulatory mechanism of MT in *A. mongolicum* seedlings under drought stress was revealed. Physiological analysis showed that MT alleviated drought stress by increasing chlorophyll content and enhancing antioxidant defense activity to reduce cell membrane damage and ROS accumulation. The integrated transcriptomic and metabolomic analysis showed that MT responds to drought stress mainly by regulating DEGs and DEMs in flavonoid biosynthesis and carbohydrate metabolism pathways. Our findings provide a basis for a further comprehensive and systematic analysis of the molecular mechanism by which MT alleviates drought stress in *A. mongolicum*.

## Data availability statement

The datasets presented in this study can be found in online repositories. The names of the repository/repositories and accession number(s) can be found in the article/**Supplementary Material**.

## Author contributions

JW, BF, and SL designed the experiments and wrote the manuscript. All authors performed the experiment. JW, XG, and XW analyzed the data and prepared the figures. BF and SL provided ideas and revised the manuscript. All authors contributed to the article and approved the submitted version.
