# Supplementary material for: Exogenous melatonin ameliorates drought stress in Agropyron mongolicum by regulating flavonoid biosynthesis and carbohydrate metabolism
Source: Front Plant Sci. 2022 Dec 19;13:1051165. doi: 10.3389/fpls.2022.1051165 (PMC9806343; doi:10.3389/fpls.2022.1051165)
Supplement: Supplementary file 1 [file DataSheet_1.docx]

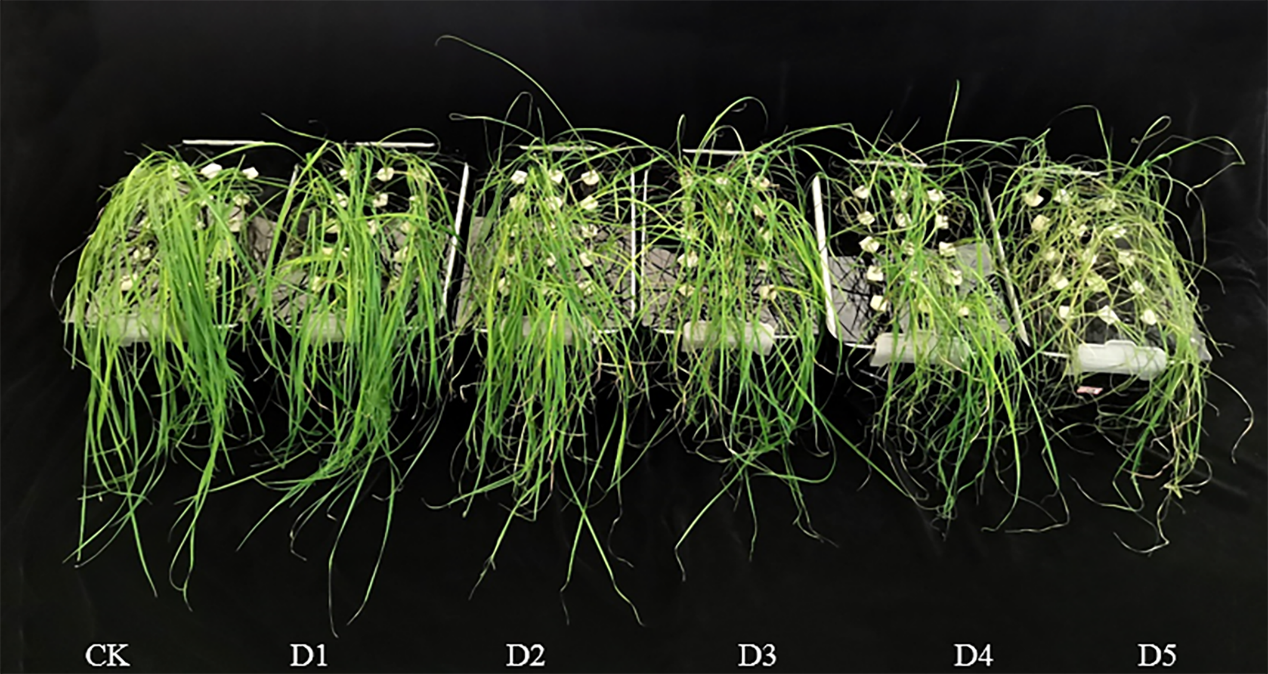


Figure S1. Phenotypic traits of *A. mongolicum* seedlings treated with different concentrations of PEG for 7 days. The different concentrations of PEG solution treatments were as follows: CK: 0% PEG; D1: 3% PEG; D2: 6% PEG; D3: 9% PEG; D4: 12% PEG; D5: 15% PEG.


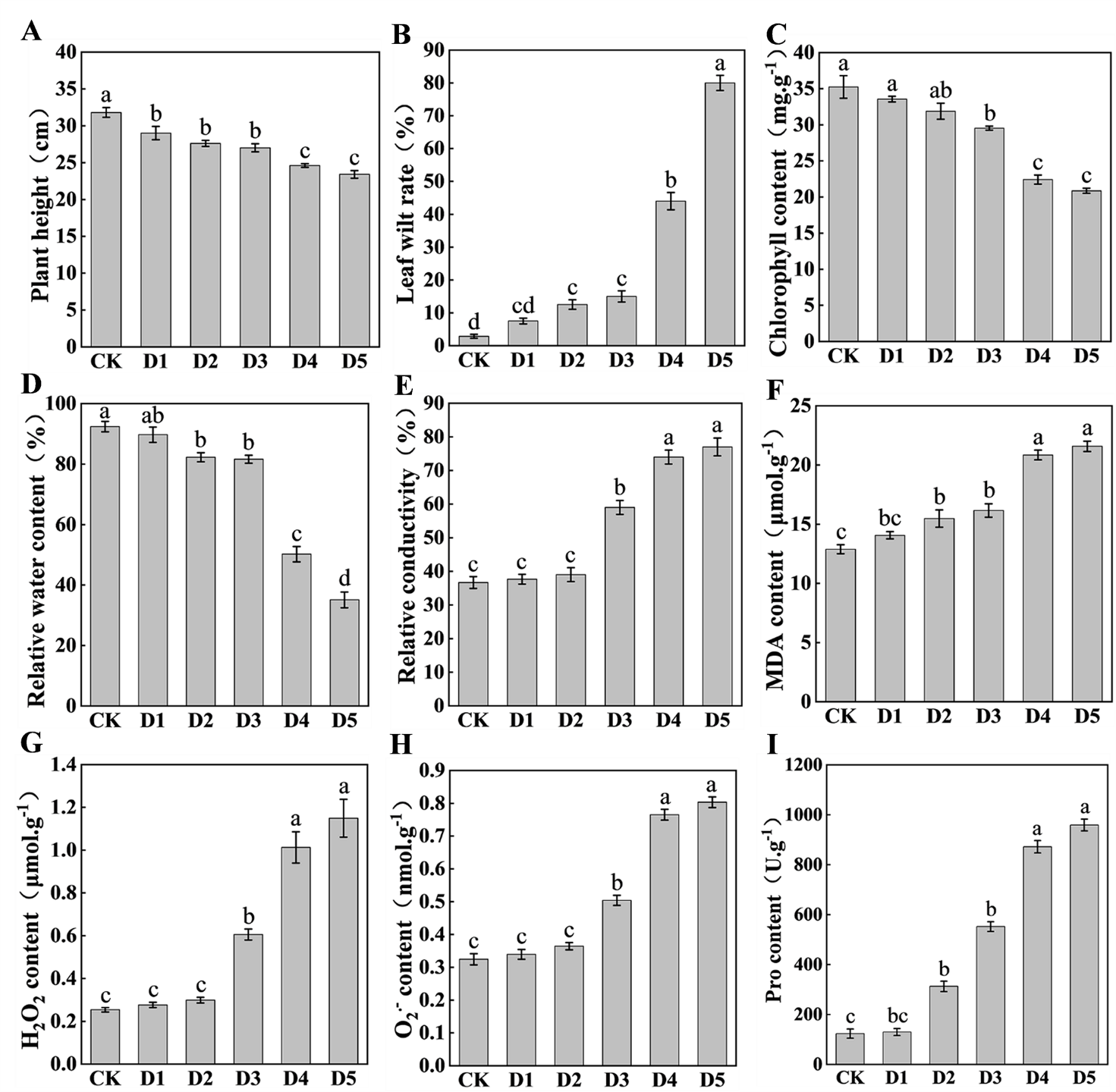


Figure S2. Physiological traits of *A. mongolicum* seedlings treated with different concentrations of PEG for 7 days. (A) plant height, (B) leaf wilt rate, (C) chlorophyll contents, (D) relative water content, (E) relative conductivity, (F) MDA content, (G) H_2_O_2_ content, (H) O_2_^.-^ content, (I) proline content. Means followed by different letters were significantly different by Duncan's test (*P* < 0.01).


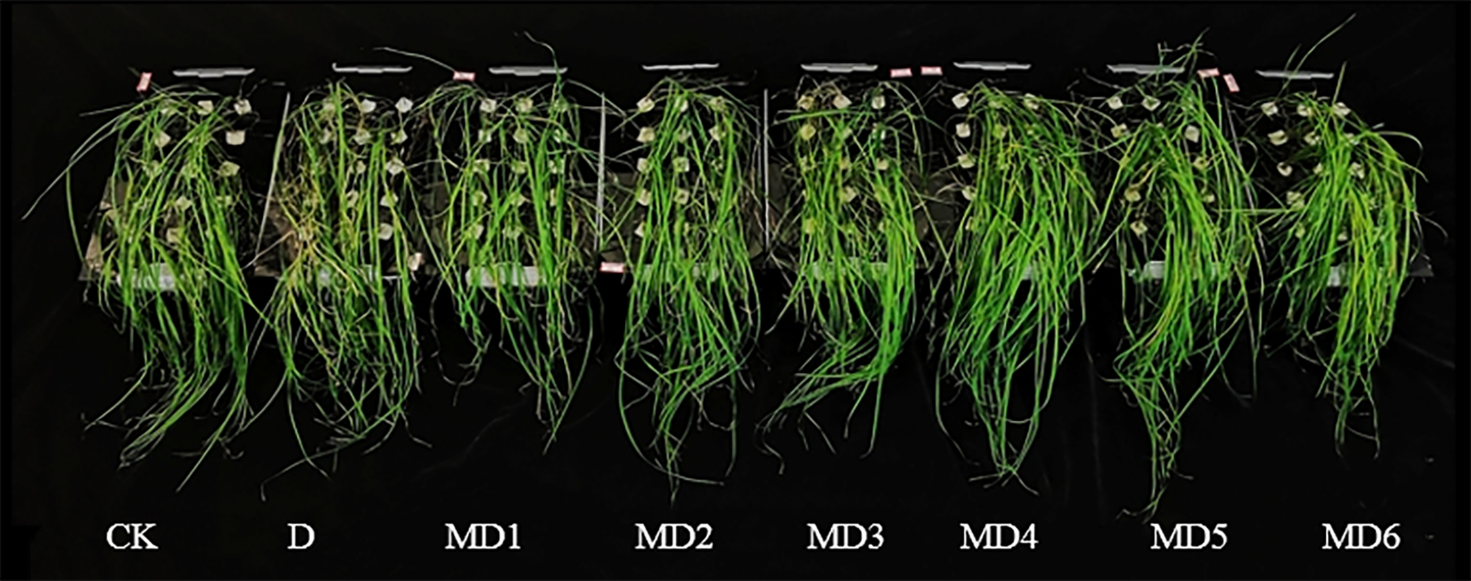


Figure S3. Phenotypic traits of *A. mongolicum* seedlings treated with different concentrations of MT under drought stress. The different concentrations of MT treatments were as follows: CK: 0% PEG; D: 12% PEG; MD1: 1 mg⋅L^-1^ MT+12% PEG; MD2: 10 mg⋅L^-1^ MT+12% PEG; MD3:50 mg⋅L^-1^ MT+12% PEG; MD4: 100 mg⋅L^-1^ MT+12% PEG; MD5: 150 mg⋅L^-1^ MT+12% PEG; MD6: 200 mg⋅L^-1^ MT+12% PEG.


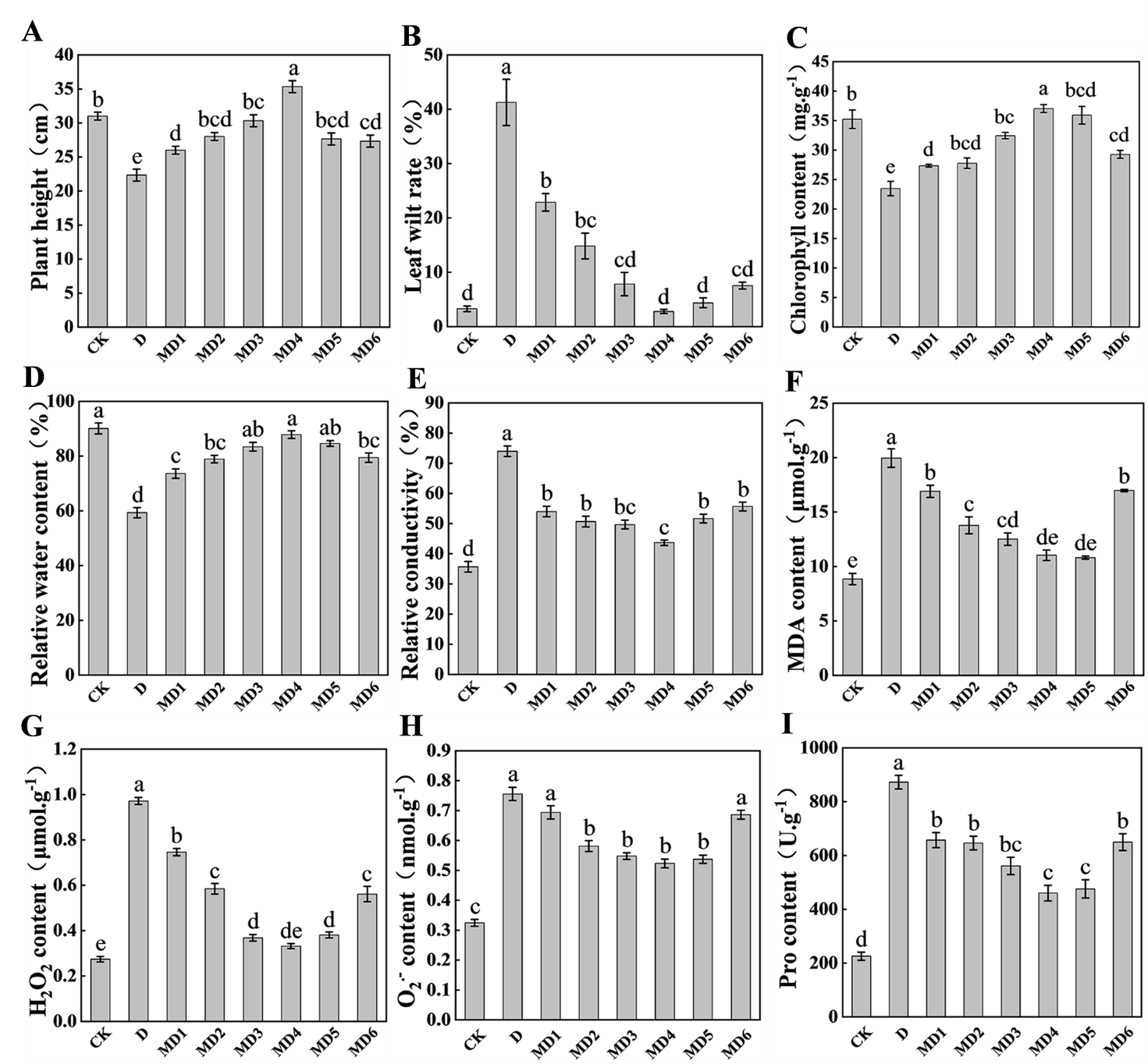


Figure S4. Physiological traits of *A. mongolicum* seedlings treated with different concentrations of MT under drought stress. (A) plant height, (B) leaf wilt rate, (C) chlorophyll contents (D) relative water content, (E) relative conductivity, (F) MDA content,(G) H_2_O_2_ content, (H) O_2_^.-^ content, (I) proline content. Means followed by different letters were significantly different by Duncan's test (*P* < 0.01).


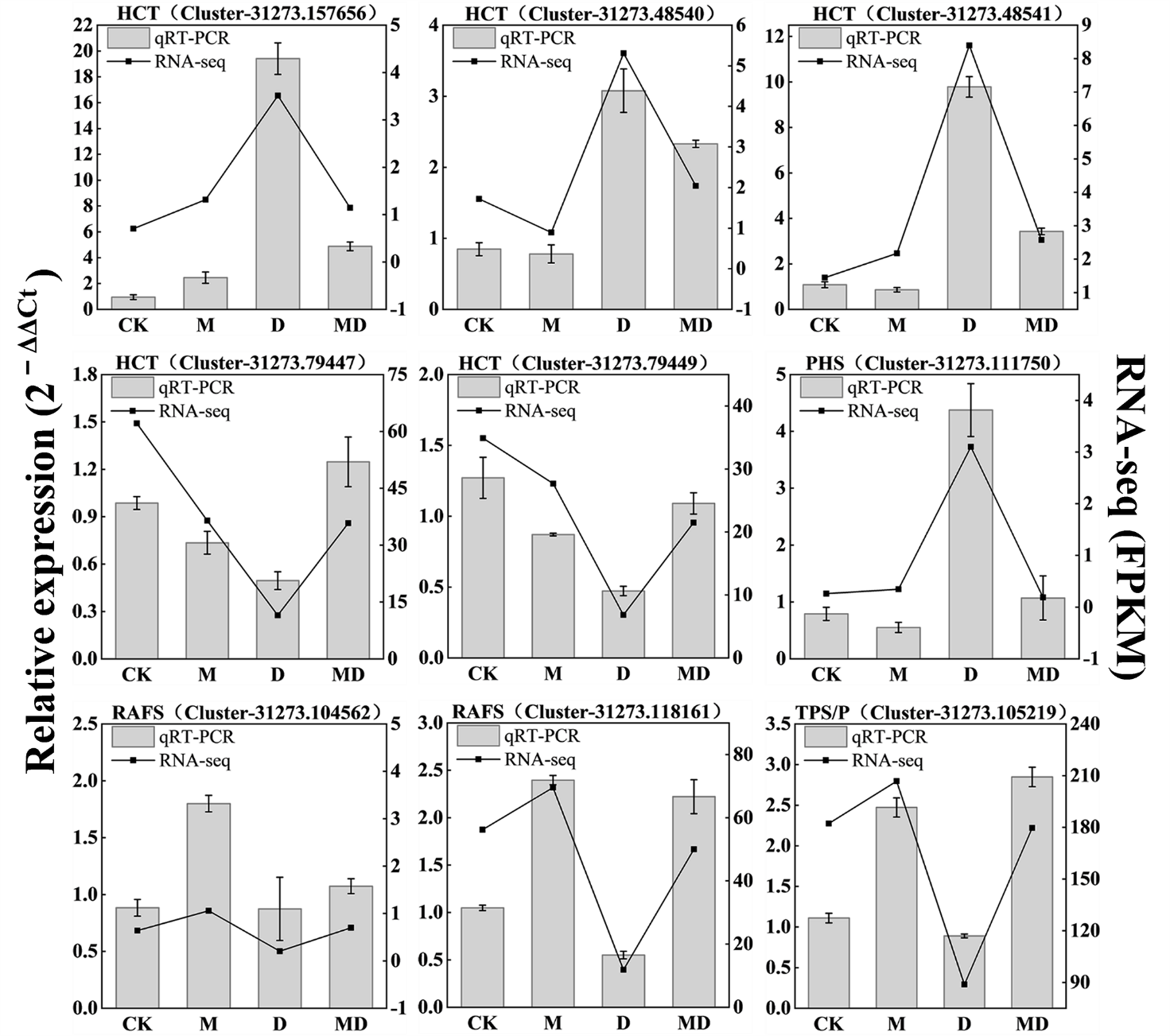


Figure S5. The qRT-PCR validation of 9 genes. HCT: shikimate O-hydroxycinnamoyltransferase; PHS: phlorizin synthase; RAFS: raffinose synthase; TPS/P: trehalose 6-phosphate synthase/phosphatase.
